# Isolation and characterization of renal cancer stem cells from patient-derived xenografts

**DOI:** 10.18632/oncotarget.6266

**Published:** 2015-11-02

**Authors:** Meriem Hasmim, Stefania Bruno, Sandy Azzi, Cindy Gallerne, Julien Giron Michel, Giulia Chiabotto, Vincent Lecoz, Cristina Romei, Grazia Maria Spaggiari, Annalisa Pezzolo, Vito Pistoia, Eric Angevin, Sophie Gad, Sophie Ferlicot, Yosra Messai, Claudine Kieda, Denis Clay, Federica Sabatini, Bernard Escudier, Giovanni Camussi, Pierre Eid, Bruno Azzarone, Salem Chouaib

**Affiliations:** ^1^ INSERM U 1186, Equipe labellisée Ligue Contre le Cancer, Gustave Roussy Campus, Villejuif, France; ^2^ INSERM UMR 1014, Lavoisier Building, Paul Brousse Hospital, Villejuif, France; ^3^ Department of Molecular Biotechnology and Healthy Science, Molecular Biotechnology Center, University of Torino, Turin, Italy; ^4^ Department of Medical Science, University of Torino, Medical School, Torino, Italy; ^5^ DIMES, UNIGE, Genova, Italy; ^6^ Laboratory of Oncology Giannina Gaslini Institute, Genoa, Italy; ^7^ Medical Oncology Department, Gustave Roussy Campus, Villejuif, France; ^8^ Laboratoire de Génétique Oncologique EPHE, Ecole Pratique des Hautes Etudes, Paris, France; ^9^ Université Paris-Sud, Assistance Publique-Hôpitaux de Paris, Service d'Anatomo-Pathologie, Hôpital Bicêtre, Le Kremlin-Bicêtre, France; ^10^ Centre de Biophysique Moléculaire, CNRS UPR 4301, Orléans, France; ^11^ INSERM UMR 972, Paul Brousse Hospital, Villejuif, France; ^12^ Stem Cell and Cell Therapy Laboratory, Istituto G. Gaslini, Genoa, Italy

**Keywords:** clear cell renal cell carcinoma, cancer stem cells, patient-derived xenografts, CD133, EpCAM

## Abstract

As rapidly developing patient-derived xenografts (PDX) could represent potential sources of cancer stem cells (CSC), we selected and characterized non-cultured PDX cell suspensions from four different renal carcinomas (RCC). Only the cell suspensions from the serial xenografts (PDX-1 and PDX-2) of an undifferentiated RCC (RCC-41) adapted to the selective CSC medium. The cell suspension derived from the original tumor specimen (RCC-41-P-0) did not adapt to the selective medium and strongly expressed CSC-like markers (CD133 and CD105) together with the non-CSC tumor marker E-cadherin. In comparison, PDX-1 and PDX-2 cells exhibited evolution in their phenotype since PDX-1 cells were CD133^high^/CD105-/Ecad^low^ and PDX-2 cells were CD133^low^/CD105-/Ecad-. Both PDX subsets expressed additional stem cell markers (CD146/CD29/OCT4/NANOG/Nestin) but still contained non-CSC tumor cells. Therefore, using different cell sorting strategies, we characterized 3 different putative CSC subsets (RCC-41-PDX-1/CD132+, RCC-41-PDX-2/CD133-/EpCAM^low^ and RCC-41-PDX-2/CD133^+^/EpCAM^bright^). In addition, transcriptomic analysis showed that RCC-41-PDX-2/CD133^−^ over-expressed the pluripotency gene ERBB4, while RCC-41-PDX-2/CD133^+^ over-expressed several tumor suppressor genes. These three CSC subsets displayed ALDH activity, formed serial spheroids and developed serial tumors in SCID mice, although RCC-41-PDX-1/CD132^+^ and RCC-41-PDX-2/CD133^+^ displayed less efficiently the above CSC properties. RCC-41-PDX-1/CD132^+^ tumors showed vessels of human origin with CSC displaying peri-vascular distribution. By contrast, RCC-41-PDX-2 originated tumors exhibiting only vessels of mouse origin without CSC peri-vascular distribution.

Altogether, our results indicate that PDX murine microenvironment promotes a continuous redesign of CSC phenotype, unmasking CSC subsets potentially present in a single RCC or generating ex novo different CSC-like subsets.

## INTRODUCTION

Current knowledge indicates that the initiation, growth, metastasis, chemo-resistance and recurrence of cancers are driven by a subset of cells endowed with stem-like properties called cancer stem cells (CSCs) or tumor- initiating cells. These cells are defined by their capability to self-renew and to recapitulate tumor formation when injected in mice [1–3]. Therefore, the identification of CSCs and a better understanding of their complex characteristics will provide very important diagnostic, therapeutic and prognostic targets for clinical application [1, 2].

Renal clear cell carcinoma (RCC) is a very aggressive cancer resistant to conventional chemo- and radiotherapy with an early metastatic evolution [4], and it seems likely that renal CSCs may have a relevant role in tumor establishment, progression, and recurrence [5]. At present, only few reports have investigated the presence of CSCs in renal carcinoma. In this context, recent histochemistry observations illustrate that the human prominin-1 (CD133) antigen, in particular of the AC133 epitope frequently employed to isolate CSCs from different tumors [6], does not seem a reliable CSC marker in RCC [7, 8]. Some groups attempted to identify renal CSCs using functional assays such as the ability to form spheres in serum-free medium [9] or the presence of the so-called Side Population [10]. Following an alternative sorting strategy, our group has recently identified, from different human RCC specimens a small subset of CSCs cells expressing the mesenchymal marker CD105 [11–13]. However, further immunohistochemistry studies on 102 RCC biopsies have shown that CD105 staining could only be detected in the cytoplasm of isolated tumor cells in the specimens derived from patients with high tumor grade and at the highest tumor stage [14]. Finally, it has been demonstrated that tumor-initiating cell frequencies were remarkably rare in well-differentiated tumors [15–17] whereas cancers in which differentiation programs are impaired would be comprised of only cells with stem and progenitor cell phenotypes [18, 19]. Overall, the above-mentioned data suggest that the current understanding of the renal stem cell system is not complete and that the kidney cancer could harbor different CSC pools displaying different phenotypes and functions according likely to tumor grade, stage and differentiation [14, 19]. Thus, it is of major interest to develop new approaches in order to identify new putative renal CSCs subsets.

In these studies, we developed an alternative strategy for identifying renal CSCs by adapting to *in vitro* culture, primary cell suspensions from serial Patient-Derived Xenografts (PDX). Of note, PDX were obtained by serially grafting tumor samples characterized according to their different degrees of differentiation, tumor stage, and aggressiveness in SCID mice [19]. Cell suspensions from PDX of four different RCC patients, characterized by the shortest latency for tumor formation in SCID mice, were chosen from the Gustave Roussy Institute cell collection. The above-mentioned cell suspensions had been immediately frozen without *in vitro* culture (P-0) or after few *in vitro* passages (P-1; P-3), representing therefore invaluable material for this type of study [19]. Only the PDX cell suspension from one (RCC-41) out of four patients was able to adapt to the selective medium growth conditions. RCC-41 is an undifferentiated RCC, and from its serial xenografts (RCC-41-PDX-1 and RCC-41-PDX-2) we isolated, sorted, and cloned three novel renal CSCs subsets that diverge from each other in phenotypic and functional properties, fulfilling however most of the criteria used to identify CSCs. These data indicate that even using PDX model, which has been reported as a necessary step for the successful isolation of renal CSCs from Wilm's xenograft [20], it is very difficult to purify CSCs from RCC. Nevertheless, our data strengthen the idea that RCC carcinomas harbor different CSC pools displaying different phenotype and functions. In addition, the serial PDX derived from a single tumor may help to unmask different CSC subsets potentially expressed by a single RCC during its *in vivo* progression, or to generate *ex novo* different CSC-like subsets.

## RESULTS

### *In vitro* selection of RCC cell suspensions derived from primary RCC xenografts in SCID mice

To test the hypothesis that patient-derived xenografts [18] could represent a source of CSCs in renal cell carcinoma, we employed never cultured or first-passage cell suspensions derived from four primary RCC xenografts. These PDXs (RCC-28-PDX-1 and -PDX-2, RCC-17-PDX-1 and -PDX-2, RCC-41-PDX-1 and -PDX-2, and RCC-47-PDX-1 and -PDX-2) characterized by different tumor stage, differentiation, histopathology and *in vivo* aggressiveness [19] (Table 1), were cultured *in vitro* with a selective medium (DMEM-LG) designed to preserve CSC stemness properties [12]. Only two cell suspensions out of eight (RCC-41-PDX-1 and -PDX-2) adapted to the selective medium and could be serially sub-cultured (Table 1). Cryopreserved cell suspensions were seeded at 5 × 10^5^ cells per 25 cm^2^ flask. RCC-41-PDX-1 and -PDX-2 cells adhered to the plastic surface with an efficiency of about 40%. After two weeks, RCC-41-PDX-1 and -PDX-2 cells started to proliferate forming isolated colonies exhibiting epithelioid morphology. Upon subculture, about 80% of RCC-41-PDX-1 and -PDX-2 cells adhered to the plastic surface, started to proliferate, and could be serially sub-cultured. Interestingly, the P-0 cell suspension derived from the original tumor (RCC-41-P-0) adapted to DMEM-LG medium but subsequently could not be serially passaged.

**Table 1:** RCC xenografts characteristics

Xenograft	Histopathology	Stage	Grade	Latency primaryXenograft (days)	Adaptation to lowG DMEM
RCC-17-PDX-1	Clear cell granular	T4N1M1	3	126	no
RCC-28-PDX-1	Clear cell granular	T3N0M0	3	92	no
RCC-41-PDX-1	Undifferentiated clear cell	T2N1M1	3	42	yes
RCC-47-PDX-1	Papillary	T3NxM0	1	76	no

### Phenotype of RCC-41-P-0 *versus* RCC-41-PDX-1 and RCC-41-PDX-2

**Figure 1:**
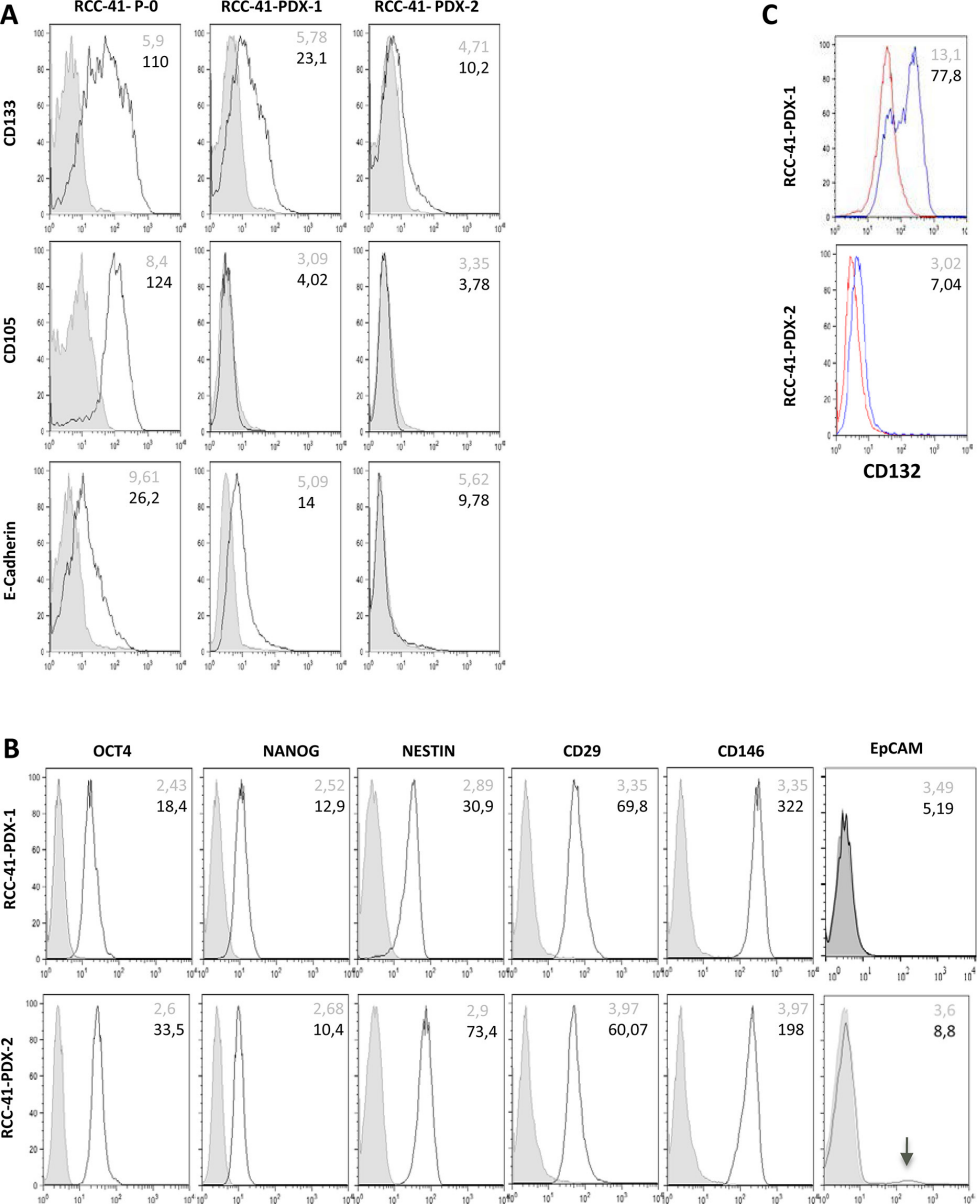
Differential expression of cell surface markers in serially xenografted RCC-41 cells **(A, B, C)** The indicated cell surface markers were analyzed in primary human cell suspension from RCC-41 tumor sample (RCC-41-P-0) and in cell suspensions from RCC-41 patient derived xenograft (RCC-41-PDX-1, RCC-41-PDX-2). Grey histograms correspond to cells incubated with the isotype-matched control antibody, and black outline histograms correspond to the analyzed markers. Grey numbers within panels correspond to Mean Fluorescence Intensity (MFI) of cells incubated with the isotype-matched control antibody, while black numbers correspond to MFI of the analyzed markers.

Flow cytometry analysis of primary RCC-41-P-0 cells shows that the majority of these cells strongly express two CSC stem-like markers: CD133 and CD105, while nearly 50% express E-cadherin (Figure 1A). The expression of E-cadherin in RCC is a good prognostic marker that indicates a tendency towards differentiation [21]. CSCs do not express differentiation markers [1–3], therefore the expression of E-cadherin suggests the persistence of a non-CSC cell fraction [12, 13] in the RCC-41-P-0 cell suspension.

Interestingly, PDX-1 and PDX-2 cell suspensions underwent a phenotypic evolution since PDX-1 displays a CD133^+^/CD105^−^/E-cad^low^ phenotype and PDX-2 a CD133^low^/CD105^−^/E-cad^−^ phenotype. These data suggest that RCC-41-PDX-1 and -PDX-2 cell suspensions could represent a mixture of CSC-like and non-CSC tumor cells. We therefore investigated the expression of additional CSC and non-CSC markers. Further phenotypic analyses (Figure 1B–1C) show that PDX-1 and PDX-2 share the expression of additional CSC markers such as CD29, CD146, OCT3/4, Nanog, and Nestin [22–24]. In contrast, they diverged in the expression of other markers displaying a bimodal distribution such as CD132 (in PDX-1, Figure 1C) and EpCAM (PDX-2, Figure 1B). EpCAM is an epithelial surface molecule whose expression in RCC is an independent predictor associated with improved survival [25], while in several cancers, EpCAM is considered as a progression/CSC marker [26]. CD132 (common gamma chain) is a cytokine receptor sub-unit expressed by normal tubular epithelial cells [27, 28] and renal CSC-CD105^+^ [11], whose loss characterizes both *in vitro* and *in vivo* non-CSC RCC cells [27, 28].

### Cell sorting and characterization of RCC-41-PDX-1 and -PDX-2 subsets

The above-mentioned results indicate that RCC-41-PDX-1 and -PDX-2 express both CSC and non-CSC markers. Therefore, we decided to enrich the CSC component by cell sorting. In RCC-41-PDX-1 cultures, we sorted to eliminate the CD132-negative cells, which likely represent the non-CSC fraction [11, 27, 28], and selected the CD132-positive cells, which could represent a CSC–like subset [11, 24, 27]. In RCC-41-PDX-2 cultures, RCC-41-PDX-2/CD133^−^ and RCC-41-PDX-2/CD133^+^ were sorted on the basis of CD133 expression. Subsequently, these sorted PDX-1 and PDX-2 subsets were cloned at limiting dilution and different clones were derived.

**Figure 2:**
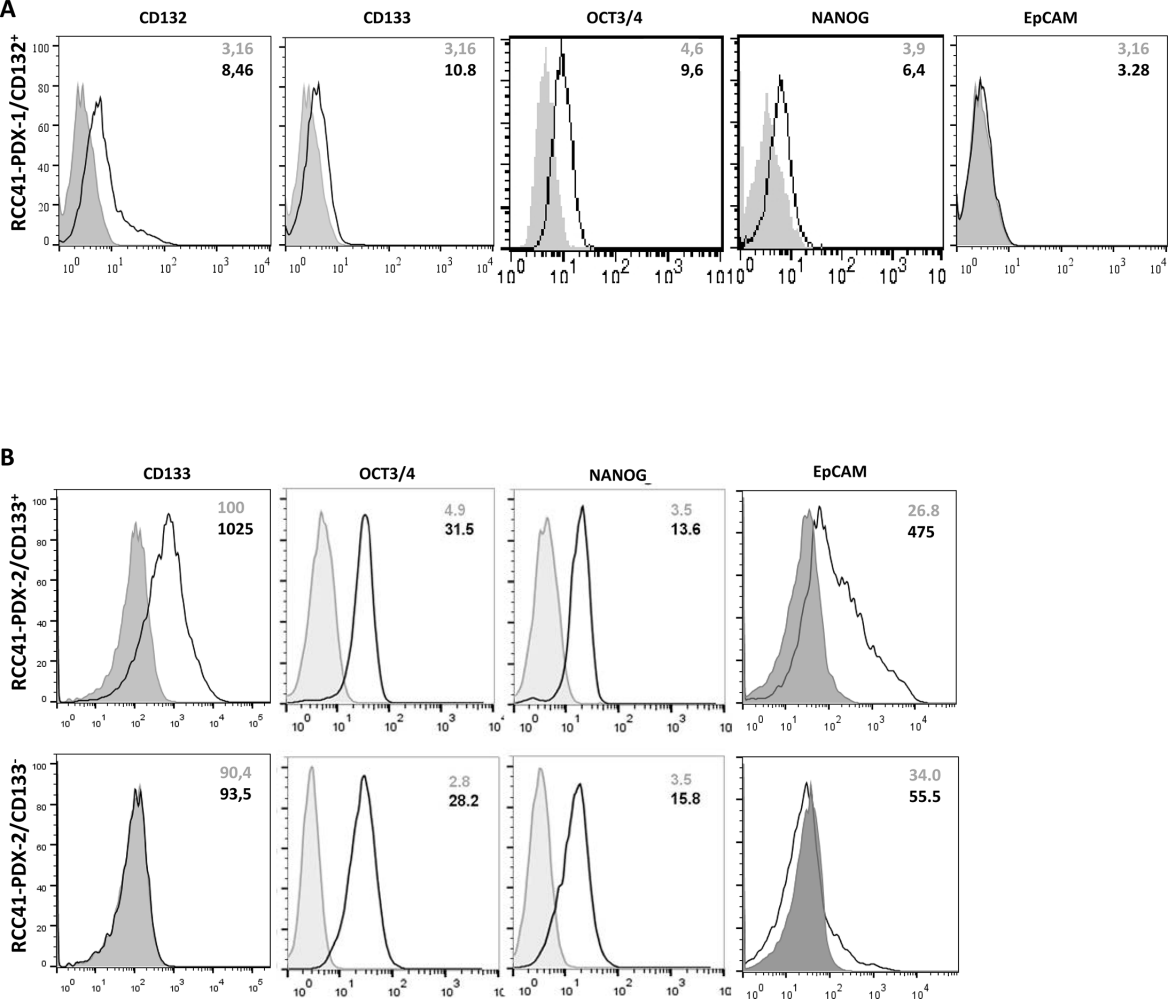
FACS analysis of stem cell and tumor markers in sorted RCC-41-PDX-1/CD132^+^, RCC-41-PDX-2/CD133^+^ and RCC-41-PDX-2/CD133^−^ cells **(A)** Expression level of CD132, CD133, OCT3/4, NANOG, and EpCAM in RCC-41-PDX-1/CD132^+^ sorted cells. Results are representative of those obtained in three experiments. **(B)** Expression level of CD133, OCT-3/4, NANOG, and EpCAM in RCC-41-PDX-2/CD133^+^ and RCC-41-PDX-2/CD133^−^ sorted cells. Results are representative of those obtained in three experiments. For **(A)** and **(B)**, grey histograms correspond to cells incubated with the isotype-matched control antibody, and black outline histograms correspond to the analyzed markers. Grey numbers within panels correspond to Mean Fluorescence Intensity (MFI) of cells incubated with the isotype-matched control antibody, while black numbers correspond to MFI of the analyzed markers.

Flow cytometry analysis shows that sorted RCC-41-PDX-1 cells expressed CD132 as a single homogeneous peak, associated with low but detectable levels of CD133, OCT3/4 and NANOG, and no expression of EpCAM (Figure 2A). Sorted RCC-41-PDX-2/CD133^−^ and RCC-41-PDX-2/CD133^+^ (Figure 2B) shared the expression of equivalent high levels of OCT3/4, NANOG but they obviously differed in the expression of CD133 and EpCAM. This latter one was very faintly expressed in RCC-41-PDX-2/CD133^−^ cells and well expressed in RCC-41-PDX-2/CD133^+^ cells.

### *In vitro* CSC functional properties of RCC-41-PDX-1 and -PDX-2 subsets

**Figure 3:**
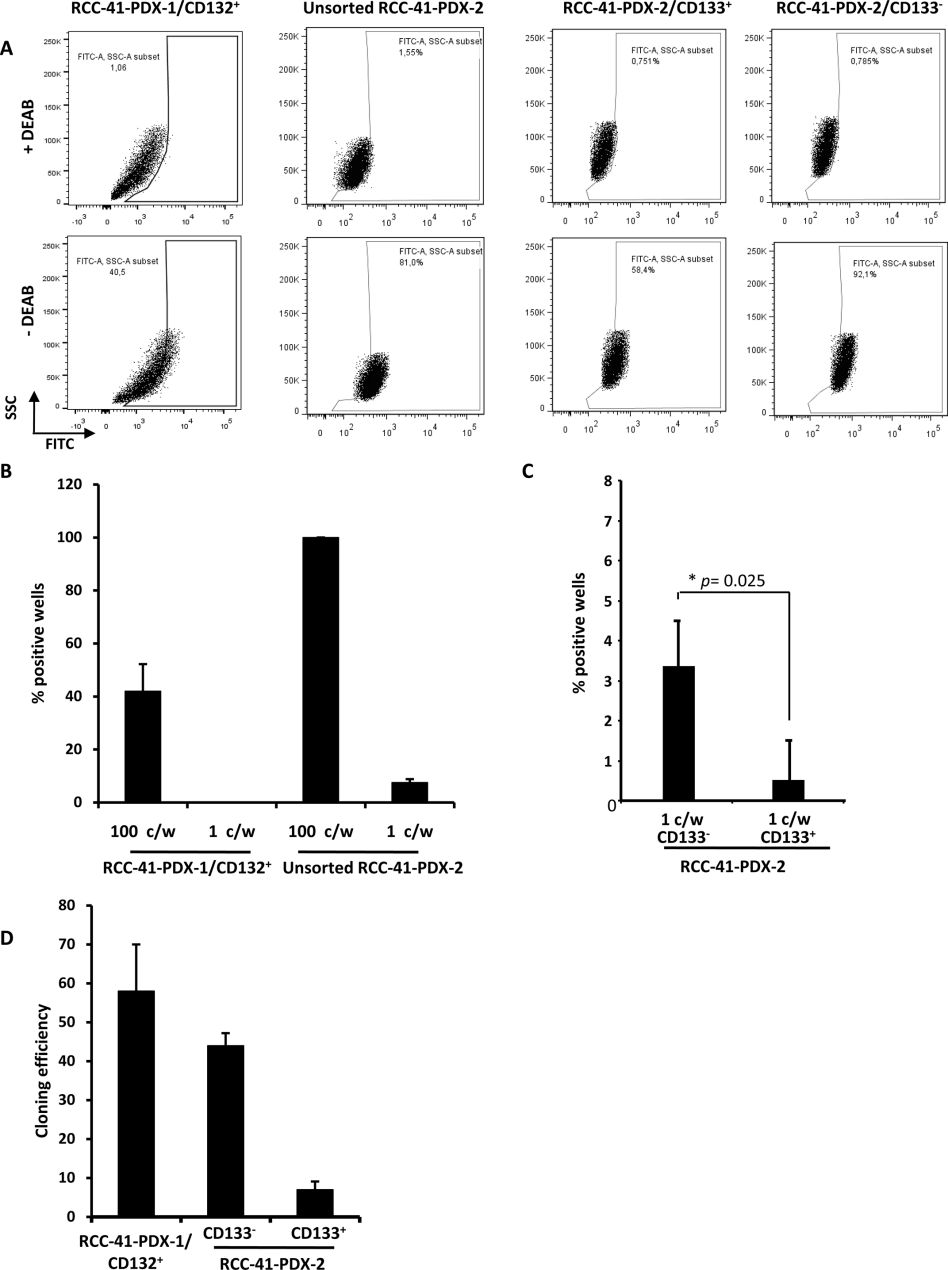
Functional CSC properties in RCC41-PDX-1/CD132^+^, unsorted RCC-41-PDX2 cells, RCC-41-PDX-2/CD133^−^, and RCC41-PDX-2/CD133^+^ subsets **(A)** The aldehyde dehydrogenase (ALDH) activity in sorted RCC-41-PDX-1/CD132^+^, unsorted RCC-41-PDX2, RCC-41-PDX-2/CD133^+^ and RCC-41-PDX-2/CD133^−^ was measured by flow cytometry (10 000 events). ALDH activity was measured in the absence and presence of the specific ALDH inhibitor DEAB following manufacturer's instructions. One experiment representative of three is shown. **(B, C)** Spheroïd formation following limiting dilution assay of the indicated cells. Data represent mean values of five replicates for two experiments; error bars correspond to 95% confidence intervals. **(D)** Clonogenic activity at limiting dilution: sorted RCC-41-PDX-1/CD132^+^, RCC-41-PDX-2/CD133^+^ and RCC-41-PDX-2/CD133^−^ were plated in the absence of serum at a density of 1 to 10 cells per well in 24-well plates containing DMEM-LG medium. After 2 weeks, each well was examined under a light microscope, and the total number of wells with colonies was determined. Data represent mean values of three replicates for three experiments; error bars correspond to 95% confidence intervals.

CSCs are more resistant to conventional chemotherapy than differentiated cancer cells. Indeed, CSCs employ several mechanisms to protect themselves against cytotoxic agents. For example, they have high levels of activity of the detoxifying enzymes ALDH, which enable them to resist the effects of chemotherapy [29]. Therefore, we measured the ALDH activity in RCC-41 by flow cytometry, using an ALDEFLUOR kit. As shown in Figure 3A, sorted RCC-41-PDX-1/CD132^+^ and RCC-41-PDX-2/CD133^+^ displayed intermediate levels of ALDH activity (40 and 60% positive cells respectively), while unsorted RCC-41-PDX-2 and sorted RCC-41-PDX-2/CD133^−^ displayed high ALDH activity (> 80% positive cells) (Figure 3A). We also investigated in the PDX-1 and PDX-2 CSC clones the presence of the most primitive cells (Side Population, SP) able to extrude the Vybrant violet dye. The experiments were performed in the presence or not of the transporter inhibitor Verapamil: no SP population could be detected in PDX-1 or PDX-2 CSC clones and PDX-1 and PDX-2 clones did not show significant phenotypic differences (data not shown). A remarkable feature of CSCs is their capability of self-renewal. Indeed this mechanism is required for preservation of the CSC pool [1–3]. In order to test their *in vitro* self-renewal potential, sorted RCC-41-PDX-1/CD132^+^ and unsorted RCC-41-PDX-2 cells were cultured at concentrations ranging from 10^2^ to 1 cell per well in DMEM-LG serum-free medium and assessed for spheroid formation (Figure 3B). Within 21 days, RCC-41-PDX-1/CD132^+^ grew forming small spheroids with an efficiency of about 40% at the limiting dilution of 10^2^ cells per well, while no spheres developed at the limiting dilution of 1 cell/well. Unsorted RCC-41-PDX-2 produced spheroids in about 100% of the wells at 10^2^ cells per well, while at the limiting dilution of 1 cell per well, RCC-41-PDX-2, sorted RCC-41-PDX-2/CD133^−^ and RCC-41-PDX-2/CD133^+^ formed spheroids with respective efficiencies of 6, 3, and 1% (Figure 3B and 3C). RCC-41-PDX-1/CD132^+^ and -PDX-2 primary spheroids were subsequently dissociated using trypsin and were able to grow serially as spheroids at their respective limiting dilutions.

Another known CSC property is the capacity to form adherent colonies at limiting dilution in serum-free medium [2, 3]. Figure 3D shows that RCC-41-PDX-1/CD132^+^ and RCC-41-PDX-2/CD133^−^ exhibit high cloning efficiency (60% and 40% respectively), while RCC-41-PDX-2/CD133^+^ display a lower cloning efficiency (5%).

### Differential expression of the multipotency gene *ERBB4* and of tumor suppressor genes in CD133-negative versus CD133-positive RCC41-PDX-2 cells

**Figure 4:**
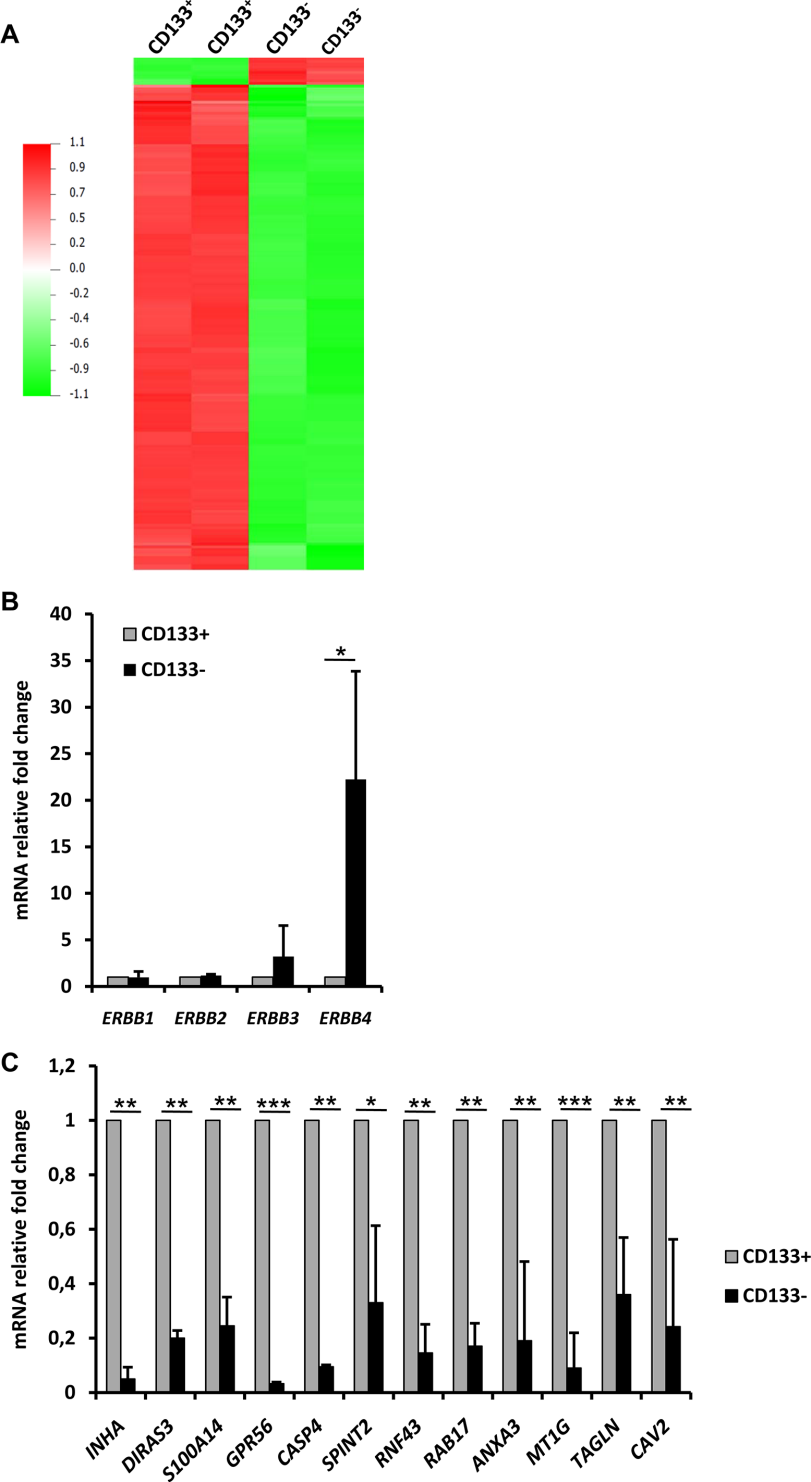
mRNA expression profiles of RCC41-PDX-2/CD133^−^ and RCC41-PDX-2/CD133^+^ subsets **(A)** Microarray analysis of CD133^+^ and CD133^−^ RCC41-PDX-2 cells. Two independent biological replicates are represented. **(B, C)** Comparative mRNA expression of *ERBB* receptors **(B)** and tumor suppressor genes **(C)** in CD133^−^ and CD133^+^ RCC41-PDX-2 cells by RT-qPCR. Presented results are mean of three independent experiments with *p < 0.05, **p < 0.005, and ***p < 0.0005.

To better understand the growing advantage of RCC41-PDX-2/CD133^−^ cells over the RCC41-PDX-2/CD133^+^ cells, we performed microarray analysis. 486 genes were differentially expressed with more than 2-fold change and an adjusted *p*-value of 0.05 (Figure 4A). Among them, 63 genes were up-regulated and 423 were down-regulated in RCC-41-PDX-2/CD133^−^ versus RCC-41-PDX-2/CD133^+^. We first analyzed genes encoding cell surface molecules and found that CD133^−^ cells over-expressed the EGFR family member *ERBB4* as compared to CD133^+^ cells, which was confirmed by qPCR (more than 20 fold) (Figure 4B). The other family members of the ERBB receptors (*ERBB1*, *ERBB2*, *ERBB3*) were not differentially expressed, suggesting a selective up-regulation of *ERBB4* (Figure 4B). Interestingly, CD133^+^ cells displayed by RT-qPCR an increased expression for potential tumor suppressor genes such as *INHBA* [30, 31], *DIRAS3* [32], *S100A14* [33, 34], *GPR56* [35] [35], *SPINT2* [36, 37], *RNF43* [21, 22, 38–40], *MT1G* [23–25, 41], *TAGLN* [42] [26, 42], *CAV2* [43, 44] (Figure 4C). Among these genes, *INHBA* [30], *MT1G* [41] and *SPINT2* [37] were reported to have tumor suppressive functions in renal cancers. As well, *ANXA3*, whose decrease was associated with renal cancer development, was also augmented [45]. The pro-apoptotic gene *CASP4* [46] was also found to be up-regulated (Figure 4C).

### Tumorigenic potential of human renal RCC-41-PDX-1/CD132^+^ in SCID mice

**Figure 5:**
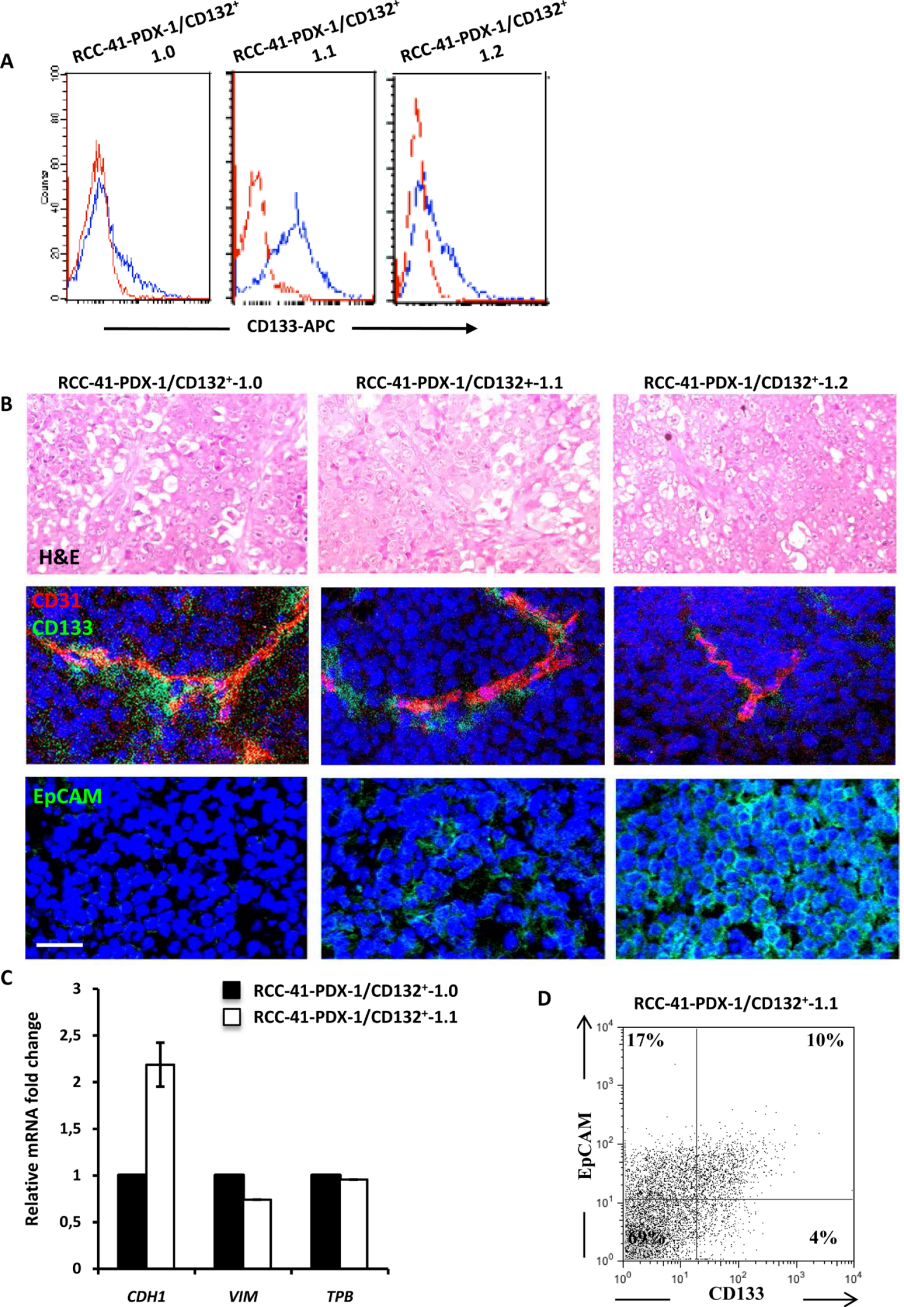
Tumorigenic potential of human renal RCC-41-PDX-1/CD132^+^ in SCID mice **(A)** Flow cytometry analysis of CD133 expression in RCC-41-PDX-1/CD132^+^ cell suspensions recovered from serial xenografts in SCID mice. Red outline histograms correspond to analyzed markers, and blue outline histograms correspond to cells incubated with the isotype-matched control antibody. **(B)** Vessel staining (CD31) and serial tumor formation by RCC-41-PDX-1/CD132^+^ cells subcutaneously injected in SCID mice. *Top row*: Representative hematoxylin and eosin staining showing the morphological appearance of an undifferentiated renal carcinoma with rare clear cells. *Middle row*: Representative immunohistochemistry of serial tumor sections showing positivity for human CD31 using a mAb that did not cross-react with the mouse CD31 (tumor vessels) and human CD133. CD133^+^ cells show a typical peri-vascular distribution. *Bottom row*: Representative immunohistochemistry of serial tumor sections showing positivity for human EpCAM. Original view × 40 (top) and × 60 (middle and bottom). Data are representative of 3 experiments with similar results. **(C)** Quantitative PCR analysis of E-cadherin (*CDH1*) and Vimentin (*VIM*), expression in RCC-41-PDX-1/CD132^+^ cells. Data were normalized using TATA binding gene mRNA (TBP), as endogenous control. **(D)** Flow cytometry analysis of CD133 and EpCAM expression in RCC-41/PDX-1/CD132^+^-1.1 cell suspension recovered from serial xenografts in SCID mice.

The results depicted in Table 2 shows that subcutaneous injection of 10^3^ RCC-41-PDX-1/CD132^+^-1.0 cells into SCID mice induced the formation of tumors within 3 weeks in all studied mice (*n* = 8 mice), while subcutaneous injection of 10^2^ RCC-41-PDX-1/CD132^+^-1.0 induced the formation of tumors in 2 out of 8 mice. RCC-41-PDX-1/CD132^+^-1.0 xenografts were enzymatically dissociated and the derived cell suspension (RCC-41-PDX-1/CD132^+^-1.1) was phenotypically characterized and subsequently re-injected in SCID mice at 10^3^ and 10^2^ cells per mouse. RCC-41-PDX-1/CD132^+^-1.1 induced the formation of tumors within 3 weeks in 5 out of 6 mice at both cell concentrations, indicating the acquisition of an increased tumor forming efficiency at the lower cell concentration. RCC-41-PDX-1/CD132^+^-1.1 xenografts subjected to the same enzymatic dissociation procedure produced the derived cell suspension termed RCC-41-PDX-1/CD132^+^-1.2. These cells exhibited a highly decreased capacity to form tumors in the SCID mice: after 3 weeks only 1 out of 6 mice developed a tumor and only at the concentration of 10^3^ cells. These data show that in SCID mice, RCC-41-PDX-1/CD132^+^ generates serially transplantable tumors with a variable efficiency. The important differences in the tumor-forming ability observed in the serial RCC-41-PDX-1/CD132^+^ xenografts led us to investigate whether these variations could be associated with a modified expression of CSC markers. For this purpose, we analyzed the phenotypes of all injected cell suspensions. Flow cytometry analysis indicates that the percentage of CD133^+^ cells present in the cell suspension injected in the SCID mice directly correlated to the tumor forming ability of the cells (Figure 5A) suggesting therefore that, contrary to what previously reported [7, 8], CD133 may help to identify subsets of CSCs in RCC.

**Table 2:** Serial tumor formation of RCC-41-PDX-1/CD132^+^ cells in SCID mice as a function of number of injected cells

	Number of injected cells per mouse (*n* = 6 to 8)	Number of mice with tumors (Delay 3 weeks)
**RCC-41-PDX-1/CD132^+^-1.0**	10^2^	2/8
	10^3^	8/8
**RCC-41-PDX-1/CD132^+^-1.1**	10^2^	5/6
	10^3^	5/6
**RCC-41-PDX-1/CD132^+^-1.2**	10^2^	0/6
	10^3^	1/6

### Progressive epithelial differentiation and presence of human vessels in RCC-41-PDX-1 serial tumors

Histologic analysis of tumors serially generated in SCID mice (RCC-41-PDX-1/CD132^+^-1.0, −1.1 and −1.2) shows a homogenous histopathology consisting of highly mitotic undifferentiated carcinomas, and rare clear cells (Figure 5B). Co-staining with anti-hCD31 and anti-hCD133 antibodies shows that several vessels of human origin are detectable in the serial xenografts (Figure 5B) and that CD133^+^ cells display a peri-vascular distribution (Figure 5B) that is maintained in the serial xenografts even if their frequency progressively decreases. In serial xenografts starting from PDX-1.1, we observed the appearance of cells expressing the human epithelial marker EpCAM (Figure 5B), which strongly increases in PDX-1.2, suggesting a natural tendency towards epithelial differentiation, that is associated with a rarefied frequency of CD133^+^ cells (Figure 5A and 5B). Thus, RCC-41-PDX-1/CD132^+^ cells are able to generate serially transplantable tumors in SCID mice, which recapitulate the histopathology of the original RCC and show a tendency for a progressive engagement in an epithelial differentiation pathway. This was confirmed by the increased expression of E-cadherin and the decreased expression of vimentin analyzed by RT-qPCR (Figure 5C). Interestingly, flow cytometry analysis performed on RCC-41-PDX-1/CD132^+^-1.1 tumor cell suspensions reveals the presence of three subsets: a first subset of CD133^−^/EpCAM^+^ (17%) represents a fraction of non-CSC/RCCs, while two smaller subsets of CD133^+^/EpCAM^−^ (4%) and CD133^+^/EpCAM^+^ (10%) cells (Figure 5D) likely represent RCC-41 CSCs differing for the acquisition of EpCAM [26].

### Tumorigenic potential of human renal unsorted RCC-41-PDX-2 and RCC-41-PDX-2 subsets in SCID Mice

**Figure 6:**
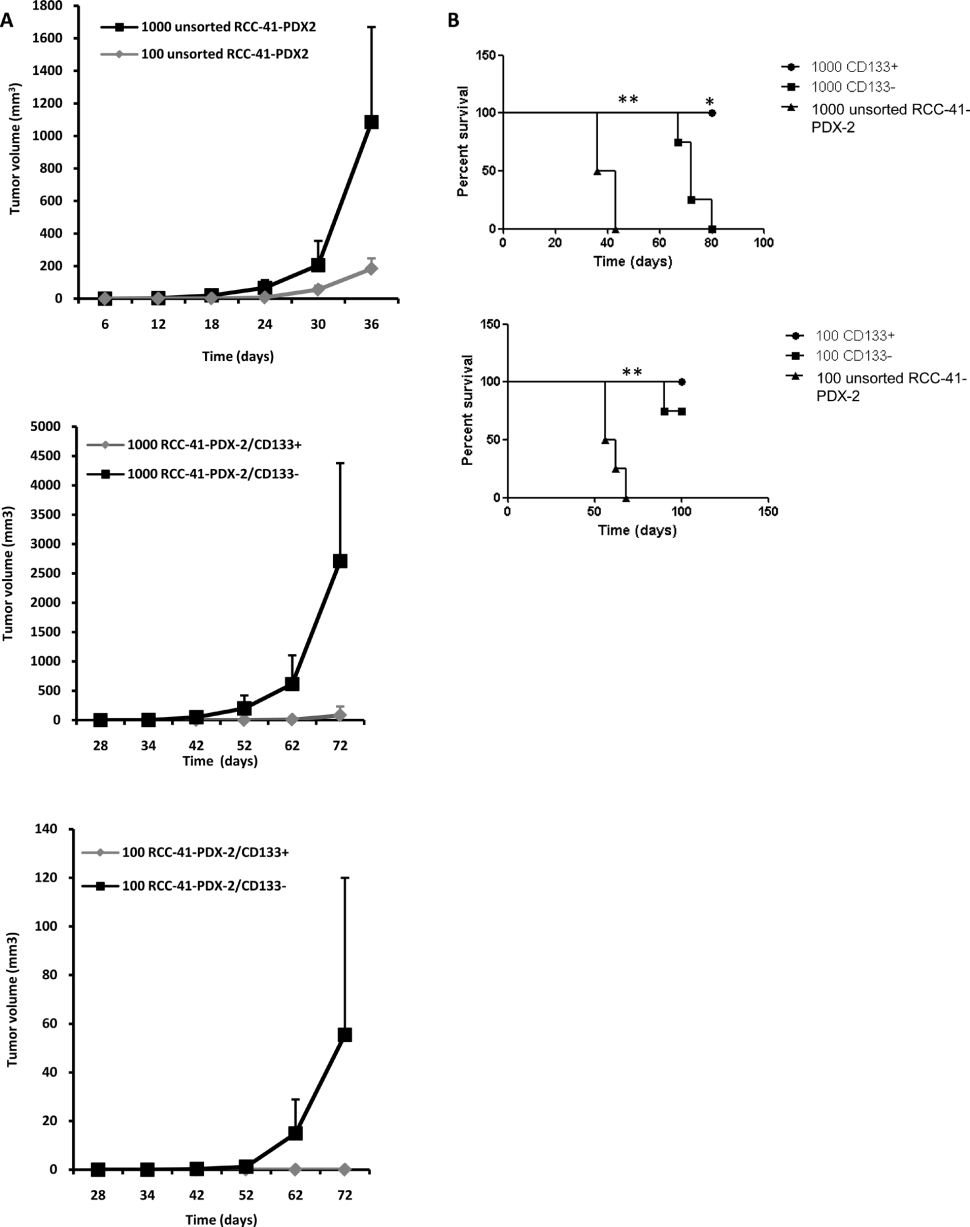
Tumorigenic potential of human renal RCC-41-PDX-2 and RCC-41-PDX-2 subsets in SCID mice **(A)** Tumor formation of unsorted RCC-41-PDX-2, and freshly sorted RCC-41-PDX-2/CD133^−^ and RCC-41-PDX-2/CD133^+^ cells in SCID mice as a function of number of injected cells 10^2^ and 10^3^ cells of each cell subtype were subcutaneously injected into SCID mice (*n* = 4 per group). Represented is the time for evaluation of tumor volume (mm^3^). Data are representative of 3 independent experiments with similar results. **(B)** Representation of tumor growth rate using survival curve. Death corresponds to a xenografted tumor reaching 1500 mm^3^. Data are representative of 3 independent experiments with similar results. *and **in (C) indicates respectively *p* < 0.05 and *p* < 0.005 by the log-rank test.

Subcutaneous injection of 10^3^ and 10^2^ RCC-41-PDX-2 and RCC-41-PDX-2/CD133^−^ cells into SCID mice generated tumors within 3 weeks (*n* = 4) (Table 3 and Figure 6A and 6B). RCC-41-PDX-2-generated tumors grew twice as fast as RCC-41-PDX-2/CD133^−^ tumors in a statistically significant way (Figure 6A and 6B). Indeed, RCC41-PDX-2-injected mice developed large tumors (1500 mm^3^) within 4 weeks of injection whereas mice engrafted with RCC41-PDX-2/CD133^−^ cells reached this volume after 8 weeks (Figure 6A and 6B). By contrast, RCC-41-PDX-2/CD133^+^ subset exhibited a consistent reduced tumor forming efficiency as compared to RCC41-PDX-2 and RCC-41-PDX-2/CD133^−^. Indeed, RCC-41-PDX-2/CD133^+^ formed smaller palpable tumors (100 mm^3^) detected in the injected mice after 9 weeks and only at the concentration of 10^3^ cells per mouse (Figure 6). This differential tumor growth was statistically significant, and indicated that the RCC41-PDX-2/CD133^−^ subset displays a higher tumorigenic potential than the CD133^+^ subset.

**Table 3:** Tumor formation of unsorted RCC-41-PDX-2, and sorted RCC-41-PDX-2/CD133^+^ and RCC-41-PDX-2/CD133^−^ cells in SCID mice as a function of number of injected cells (*n* = 4 per group)

	Number of injected cells per mouse (*n* = 4)	Number of mice with tumor (Delay 3 weeks)	Number of mice with tumor (Delay 6 weeks)	Number of mice with tumor (Delay 9 weeks)	Number of mice with tumor (Delay 10 weeks)
**Unsorted RCC-41-PDX-2**	10^2^	3/4	4/4	-	-
	10^3^	4/4	4/4	-	-
**RCC-41-PDX-2/CD133^+^**	10^2^	0/4	0/4	0/4	0/4
	10^3^	0/4	0/4	4/4	4/4
**RCC-41-PDX-2/CD133^−^**	10^2^	2/4	2/4	2/4	4/4
	10^3^	3/4	4/4	4/4	4/4

### Loss of human endothelial micro-vessels (EM) in RCC-41-PDX-2 tumors

**Figure 7:**
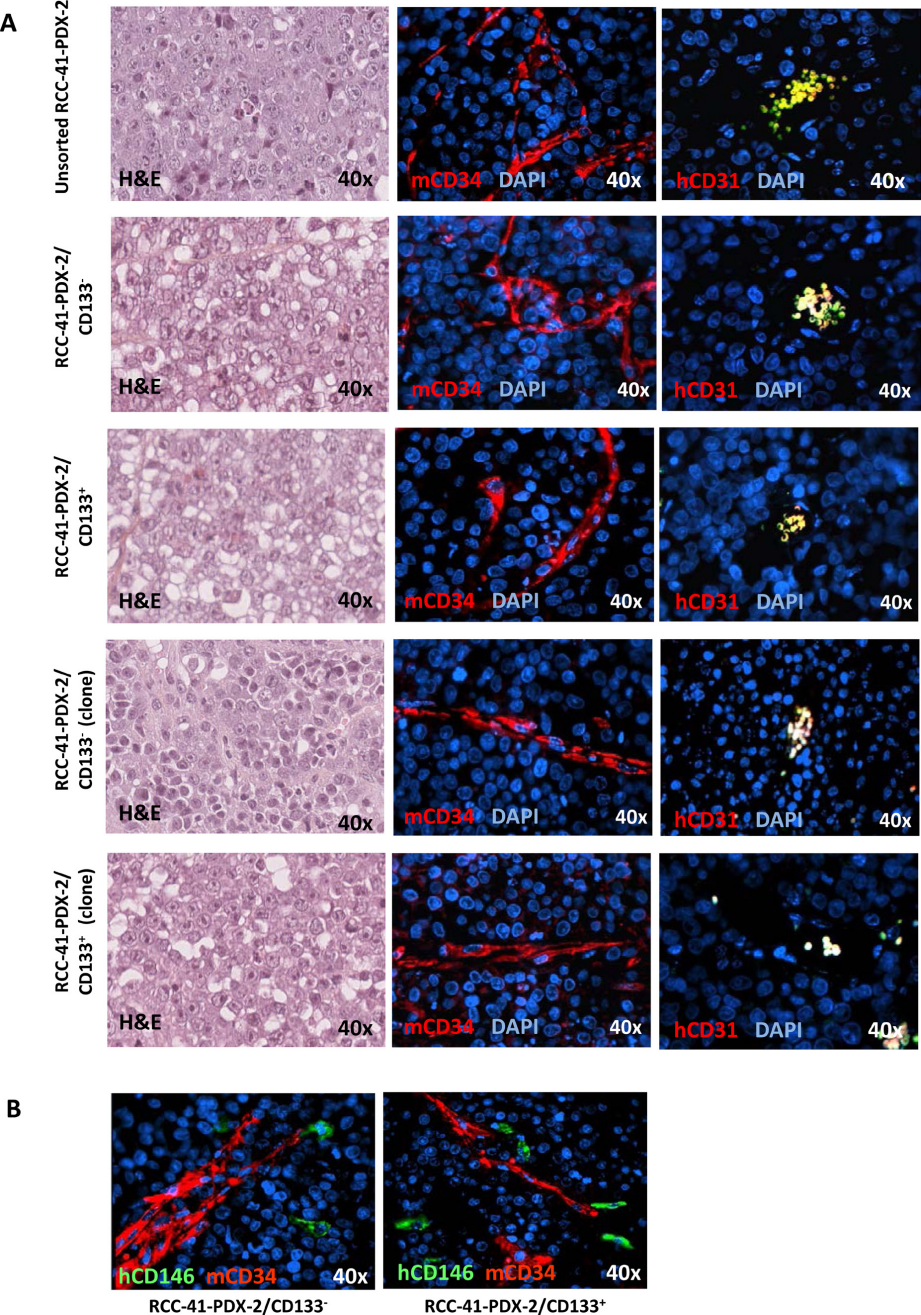
Vessel staining in human renal unsorted RCC-41-PDX-2 and RCC-41-PDX-2 cell subsets subcutaneously injected in SCID mice **(A)** Representative hematoxylin/eosin and immunofluorescence stainings of serial tumor sections showing positivity for mouse CD34 but not for human CD31 antibodies in RCC-41/PDX-2-derived xenografts. **(B)** Representative immunofluorescence stainings of serial tumor sections showing positivity for mouse CD34 and human CD146 antibodies in RCC-41/PDX-2 subsets.

H&E staining of tumors generated in SCID mice by RCC-41-PDX-2, and RCC-41-PDX-2/CD133^−^, RCC-41-PDX-2/CD133^+^ and their respective clones, shows that PDX-2 xenografts exhibit histopathology similar to that observed in PDX-1 tumors, consisting of highly mitotic undifferentiated carcinomas, and rare clear cells (Figure 7A). Immunofluorescence analysis of tumor vessels shows off a remarkable property that distinguishes RCC-41-PDX-1/CD132^+^ from RCC-41-PDX-2 xenografts: in all PDX-2 xenografts, vessels are entirely of murine origin as shown by EM counts using hCD31 and mCD34 mAbs proved to be rigorously species-specific in a previous study [47]. All vessels present in PDX-2 xenografts were lined with mCD34^+^ endothelial cells, whereas vessels lined with hCD31^+^ endothelial cells were never detected (Figure 7A). Moreover PDX-2/CSCs, identified *in vivo* through the expression of CD146, were detected nearby murine vessels but did not show the strict peri-vascular distribution detected in PDX-1 xenografts (Figure 7B).

## DISCUSSION

The identification of renal CSCs represents a therapeutic priority that, for the moment, remains somewhat elusive due to RCC genetic and histological heterogeneity [4, 5]. Recently, a CSC population expressing CD105 was purified from RCC patients [12]. An immunohistochemical study, performed on paraffin-embedded tumor samples derived from 102 RCC patients, showed that CD105 expression in tumor cells was found in high-grade tumors and highest tumor stages [14]. This would suggest that RCC may harbor CSCs with different phenotypes [5]. Thus we developed an alternative strategy for identifying putative renal CSCs, different from the CD105^+^ cell sorting [12]. For this purpose, we exploited the Gustave Roussy Institute cell bank; using never-cultured cell suspensions derived from the four more aggressive serial RCC-PDX displaying different phenotypes, tumor stage and grade [19, 48]. Only RCC-41-PDX-1 and -PDX-2 cell suspensions out of four different RCC-PDX adapted to the selective medium conceived for preserving *in vitro* the stem cell properties [12]. Interestingly, we also tried to culture the primary cell suspension derived from the original tumor (RCC-41-P-0); however, while the cells attached and grew slowly up to near confluence, they could not be further passaged.

Comparison of the phenotype of RCC41-P-0, RCC-41-PDX-1 and RCC-41-PDX-2 primary cultures showed major phenotypic differences. Indeed, RCC-41-P-0 cells expressed a CD133^bright^/CD105^brigh^/E-Cadherin^+^ phenotype. The unusual high expression of the stemness markers CD133 and CD105 is probably related to the fact that RCC-41 is an undifferentiated tumor [48] and points out the likely existence in RCC-41-P-0 of a large proportion of very primitive CSC-like cells, while the expression of E-cadherin supports the presence of differentiated non-CSC tumor cells [21, 49, 50]. By contrast, RCC-41-PDX-1 expressed a CD105^−^/CD133^+^/E-cadherin^low^ phenotype while RCC-41-PDX-2 were CD105^−^/CD133^low^/E-cad^−^. In order to further enrich RCC-41-PDX-1 and RCC-41-PDX-2 subsets in CSC, we developed different cell sorting strategies. Sorted cells were cultured and amplified over two *in vitro* passages, analyzed for their phenotype, and subsequently cloned at limiting dilution. Using the PDX model, we selected three potential CSCs subsets that could be further distinguished on the basis of the expression of the CSC-like marker EpCAM [26]: CSC/PDX-1/CD132^+^/EpCAM^−^, CSC/PDX-2/CD133^−^/EpCAM^low^, and CSC/PDX-2/CD133^+^/EpCAM^bright^.

The sorted CSC/PDX-1 and CSC/PDX-2 subsets, as well as their respective clones, display properties typical of CSCs detected in other solid tumors. Namely they: 1) express stem cell markers, 2) exhibit clonal growth at limiting dilution, 3) grow and can be serially transferred as floating spheroids, 4*)* express mid-high levels of the detoxifying enzyme ALDH, a typical CSC marker [51], and 5) generate serially transplantable tumors characterized by similar histopathology. However it must be stated that serial PDX-1/CD132^+^ xenografts displayed microvessels of human origin and strict peri-vascular distribution of CSCs that favors the preservation of stemness [52], while PDX-2 xenografts exhibited microvessels of murine origin without peri-vascular distribution of CSCs.

In addition, between PDX-1/CD132^+^-1.1 and PDX-1/CD132^+^-1.2 xenografts, we observed a decreased frequency of CD133^+^ CSCs while an increasing fraction of tumor cells expressed EpCAM. More precisely, double staining performed on PDX-1/CD132^+^-1.1 cell suspensions indicates the presence of three different subpopulations: i) PDX-1/CD132^+^/CD133^−^/EpCAM^+^ cells, ii) PDX-1/CD132^+^/CD133^+^/EpCAM^−^ cells and iii) PDX-1/CD132^+^/CD133^+^/EpCAM^+^ cells. Thus, we propose that serial xenografts of PDX-1/CD132^+^ in SCID mice progressively produce differentiated tumors, as shown by the increased expression of E-cadherin, the absence of vimentin expression, and the massive appearance of CD133^−^/EPCAM^+^ cells, that in PDX-1/CD132^+^-1.2 xenografts constituted more than 70% of the non-CSC tumor cell bulk. On the other hand, the PDX-1/CD132^+^/CD133^+^/EpCAM^−^ cells represent the original CSC/PDX-1/CD132^+^ subset, while the PDX-1/CD132^+^/CD133^+^/EpCAM^+^ cells probably represent a new emerging majoritary CSC subset.

Finally, in PDX-2 xenografts, murine microenvironment induces a remodeling of the CSC properties favoring the appearance of two novel subsets of less primitive CSC since they have lost the potential to generate human microvessels and are characterized by different phenotypes: PDX-2/CD133^−^/EpCAM^−^/ERBB4^+^ is the predominant aggressive subset while the PDX-2/CD133^+^/EpCAM^bright^ is a minor, less aggressive CSC subset characterized by the expression of several tumor suppressor genes.

In conclusion, our data support the idea that the serial PDX derived from a single tumor may help to unmask the different CSC subsets potentially expressed by a single RCC during its *in vivo* progression. Alternatively, it is also possible to postulate that the PDX CSC niche, which is of murine origin, could redesign the properties of human CSC favoring the generation of modified CSCs that become unable to generate human microvessels hampering somehow the development of targeted anti-angiogenic preclinical approaches.

## MATERIALS AND METHODS

### Cell culture

Cell suspensions from 4 different RCC-PDX xenografts (RCC-17-PDX-1 and -PDX-2, RCC-28-PDX-1 and -PDX-2, RCC-41-PDX-1 and -PDX-2, RCC-47-PDX-1 and -PDX-2) that resulted in tumor development in SCID mice [19] and RCC-41-P-0 were adapted to *in vitro* culture using a multipotent adult progenitor cell medium consisting of DMEM-LG (Invitrogen, Paisly, UK) with insulin-transferrin selenium 1x (Invitrogen, Paisley, UK), 10^−9^ M dexamethasone, 100 *U* penicillin, 1000 *U* streptomycin, 10 ng/ml epidermal growth factor (EGF) (Sigma-Aldrich) and 5% FCS (Euro Clone, Wetherby, UK). Adherent cells were maintained in complete DMEM-LG for more than 30 passages, with no change in the expression of stem cell markers as determined by FACS analysis.

### Antibodies, cytokines, and reagents

Conjugated antibodies against NANOG (phycoerythrin (PE) conjugated goat polyclonal (IC1997P), 1:200 dilution), OCT-3/4 (PE conjugated goat polyclonal (IC1759P), 1:200 dilution), and Nestin (PE conjugated mouse monoclonal (IC1259P), 1:200 dilution) were purchased from R&D Systems Europe Ltd (Abingdon, UK). Fluorescein isothiocyanate (FITC)-conjugated anti-CD90 (mouse monoclonal, 1:100 dilution) was purchased from Dianova GmbH (Hamburg, Germany).

All other antibodies used for cytofluorimetric analyses (FACS) were directly conjugated with FITC, PE, or APC: anti-CD31, antiCD-146/Muc-18, anti- CD29, anti-CD105, and anti-CD133 monoclonal antibodies (mAbs) (all from Miltenyi Biotec, Cologne, Germany); mAb anti-EpCAM (Biolegend, San Diego, CA). Isotype-matched FITC-, PE-, or APC- conjugated control mouse G (IgG) were from Miltenyi.

### Sphere formation assay

We evaluated the self-renewal capacity of RCC-41-PDX-1/CD132^+^, RCC-41-PDX-2, RCC-41-PDX-2/CD133^+^, and RCC-41-PDX-2/CD133^−^ by performing a limiting dilution assay for spheroid formation. Cells were plated using a FACS DiVa cell sorter equipped with autoclone software (Beckton Dickinson, Le Pont-de-Claix, France) at a density of one and 100 cells per well in ultralow attachment 96-well plates (Corning Life Sciences, Acton, MA). Each well was supplemented with 200 μl of serum-free complete DMEM-LG. After 3 weeks, each well was examined under a light microscope and the total number of wells containing spheroid colonies was determined. Five replicates were used for each condition, and the experiment was repeated two times.

### RNA extraction and quantitative real time reverse transcriptase polymerase chain reaction

Total RNA was isolated from cells using TRIZOL reagent (Invitrogen), according to manufacturer's instructions. RNA was quantified spectrophotometrically (Nanodrop ND-1000, Wilmington DE). Primers used for qRT-PCR are shown in Table 4; other primer sequences are available upon request.

**Table 4:** Primer sequences

Gene	Symbol/gene ID:	Primer sequence
E-CADHERIN	CDH1 / 999	FWD: 5′-GCATTGCCACATACACTCTCTTCT-3′ REV: 5′-GCTTGTTGTCATTCTGATCGGTTA-3′
VIMENTIN	VIM / 7431	FWD: 5′-GGAACAGCATGTCCAAATCGAT-3′ REV: 5′-CAGCAAACTTGGATTTGTACCATT-3′
TATA BINDING PROTEIN	TBP / 6908	FWD: 5′-TGTGCACAGGAGCCAAGAGT3-3′ REV: 5′-ATTTTCTTGCTGCCAGTCTGG-3′

To detect mRNA expression, first-strand cDNA was produced from 200 ng of total RNA using High Capacity cDNA Reverse Transcription Kit (Applied Biosystems, Foster City, CA). Real-time PCR experiments were performed in 20 μl reaction mixture containing 5 ng of cDNA template, the sequence-specific oligonucleotide primers (purchased from MWG-Biotech AG, Ebersberg, Germany, www.mwg-biotech.com) and the Power SYBR Green PCR Master Mix (Applied Biosystems). Negative cDNA controls (no cDNA) were cycled in parallel with each run. qRT-PCR was performed using a 96-well StepOne™ Real Time System (Applied Biosystems). mRNA comparison between samples was calculated on relative expression data normalized using TATA binding protein (TBP), as endogenous control. Fold change expression with respect to controls was calculated for all samples.

### Flow cytometry

For all assays described below, we acquired fluorescence data for 10,000 events on a FACSCalibur flow cytometer (BD Biosciences, Oxford, UK) and analyzed the data with the use of CellQuest software (BD Biosciences) or FlowJo (Treestar). The experiments were repeated at least three times. In some experiments, cells were analyzed on a FACScan (Becton Dickinson, Franklin Lakes, NJ, USA).

### Expression of cellular antigens

Expression of cell surface (E-cadherin, CD105, CD133, EpCAM, CD132 (IL-15Rγ), CD29, CD146) and intracellular (OCT-3/4, NANOG, Nestin) proteins was analyzed by flow cytometry as previously described [11, 12]. Briefly, suspensions of enzymatically- or EDTA-detached cells were permeabilized (for intracytoplasmic staining) or not (for cell surface staining) with BD Cytofix/Cytoperm 5 reagent (BD Pharmingen, Le Pont-De-Claix, France). 5 × 10^5^ cells were suspended in DMEM-LG medium supplemented with 1% FCS and stained with conjugated antibodies directed against the above-mentioned cell markers at the dilutions indicated in the “Antibodies, Cytokines, and Reagents” section. Cells were subsequently analyzed by flow cytometry.

### ALDH detection

ALDH activity was assessed by flow cytometry in RCC-41-PDX-1/CD132^+^ and -PDX-2 subsets using ALDEFLUOR kit (StemCell Technologies, Grenoble, France) in accordance with the manufacturer's instructions. Briefly, 10^6^ cells from RCC-41-PDX-1/CD132^+^ and -PDX-2 subsets were incubated with BODIPY aminoacetaldehyde, which is converted into a fluorescent molecule (BODIPY aminoacetate) in the cytoplasm. Specificity of the fluorescence was shown using the specific ALDH inhibitor diethylaminobenzaldehyde (DEAB).

### Tumorigenic potential of RCC-41-PDX-1/CD132^+^ and -PDX-2 cells in severe combined immunodeficient (SCID) mice

RCC-41-PDX-1/CD132^+^ and -PDX-2 subsets were harvested by incubation with trypsin–EDTA, washed in PBS and resuspended in 100 μL of DMEM. Aliquots of 10^2^ or 10^3^ cells were added to 100 μL of Matrigel (BD Biosciences), chilled on ice, and injected subcutaneously with a 1-mL syringe fitted with a 26-gauge needle into the left and right sides of 6-week-old male SCID mice (Charles River, Jackson Laboratories, Bar Harbor, ME). Four to eight mice were injected with each of the different RCC-41 subsets. We performed three independent experiments. At different times after cell injections (3, 6, 9, and 10 weeks) the mice were killed by carbon dioxide asphyxiation and their tumors were excised, weighed, and measured. Studies were approved by the Italian Ministry of Health and by the Institutional Review Board of the University of Turin and were performed in accordance with the National Institutes of Health Guide for the Care and Use of Laboratory Animals (Institute of Laboratory Animal Resources: 7th ed. Washington, DC, Institute of Laboratory Animal Resources, Commission on Life Sciences, National Research Council, 1996).

### Immunofluorescence

Indirect immunofluorescence was performed on formalin-fixed, paraffin-embedded tissue sections from 5 xenograft tumors as previously described [47]. Paraffin sections (4 μm thick) were processed by standard deparaffinization with xylene and hydrated in a descending ethanol series to double-distilled water. Antigen retrieval on formalin-fixed tissue section was performed using Sodium-Citrate buffer (pH 6.0). Indirect immunofluorescence was performed using the following primary antibodies: anti-human (h) CD31, monoclonal mouse (diluted 1/100; Dako Cytomation, Hamburg, Germany) and anti-mouse (m) CD34, monoclonal rat (diluted 1:100; Novus Biologicals, Littleton, CO, USA), anti-CD133 (h) (Miltenyi), and anti-EPCAM (h) (Biolegend). Slides were incubated with primary antibodies overnight at 4°C. Secondary antibodies used were: goat anti-mouse IgG Alexa-568 and goat anti-rat IgG Alexa-568 (diluted 1:200; Invitrogen, Germany). After washing, the slides were counterstained with 4′,6′-diamidino-2- phenylindole (DAPI, Sigma-Aldrich, Milan, Italy) and cover-slipped.

The endothelial micro-vessels (EM) were assessed by anti-hCD31 and anti-mCD34 staining and examination of twenty microscopic fields (0.5 mm^2^) per tumor. The most intense vascular areas (hotspots) were selected subjectively from each tumor section. The micro-vessels with a clearly defined lumen or well-defined linear vessel shape were taken into account.

For image analysis, digital images were collected using a Nikon E-1000 fluorescence microscope (Nikon Instruments, Tokyo, Japan) equipped with appropriate filter sets and the Genikon imaging system software (Nikon Instruments).

### Statistical analysis

For tumor studies in mice, the number of animals per group was based on our acquired expertise after 4 years of experiments with this model (we did not perform power calculations to determine the number of mice per group). The unit of analysis was the mouse, and the average of all tumors per mouse was taken into account. For the survival curves, the log rank test was used to compare the survival of the different groups. Otherwise, the two-sided Student's *t* test was used to compare groups. A *P* value less than .05 was considered statistically significant.
